# Factors associated with oral health service utilization among young people in southern China

**DOI:** 10.1186/s12903-024-03994-4

**Published:** 2024-02-28

**Authors:** Yunquan Cai, Shaobo Zeng, Yimei Hu, Lingfeng Xiao, Yanqing Liao, Zihui Yan, Wenxiang Zha, Junwang Gu, Qi Wang, Ming Hao, Chunmei Wu

**Affiliations:** 1 School of Public Health and Health Management, Gannan Medical University, No. 1 Yixueyuan Road, Ganzhou, Jiangxi 341000 China; 2 School of Public Health, China Medical University, Shenyang, Liaoning China; 3 Department of Public Health Surveillance, Linping Center for Disease Control and Prevention, Hangzhou, 311103 China; 4 Key Laboratory of Prevention and Treatment of Cardiovascular and Cerebrovascular Diseases of Ministry of Education, Gannan Medical University, Ganzhou, 341000 China

**Keywords:** China, Oral health service utilization, Young adults

## Abstract

**Objectives:**

To identify the patterns and influencing factors of oral health service utilization among college students, and further to provide scientific evidence for policy making on oral health education and behavioral interventions for the college population.

**Methods:**

The study population was college students in Southern China. Totally 678 students participated in the survey. A self-designed questionnaire based on Anderson’s model (predisposing factors, enabling factors, need factors) was used to survey college students. Descriptive statistics, χ^2^ test, and logistic regression were used to analyze influence factors of oral health service utilization among college students.

**Results:**

The utilization rate of oral health service in the past 12 months was 30.2%. The primary type of oral health service was treatment (59.6%), and only 12.8% were for prevention. There were 39% of the participants having oral health diseases, of which dental caries (25.7%) and oral bleeding (22.2%) were the main problems. The results from logistic regression analysis revealed that students with better beliefs (OR = 1.84, 95% CI:=1.02–3.43), frequent consumption of sugary drinks (OR = 2.90, 95% CI:=1.90–4.47), teeth brushing frequency > = 2 times per day (OR = 2.09, 95% CI = 1.24–3.61), frequent floss utilization (OR = 2.63, 95% CI = 1.21–5.76), dental caries (OR = 2.07, 95% CI = 1.35–3.17) used oral health services higher, while those lived in rural areas (OR:0.52, 95% CI = 0.34–0.80), and had only a fair concern (OR = 0.48, 95% CI = 0.31–0.74) or no concern (OR = 0.26, 95% CI = 0.08–0.67) on oral health utilized oral health services lower.

**Conclusions:**

Chinese college students demonstrate some knowledge and attitudes towards oral health. However, they tend to neglect oral hygiene and have limited understanding of their own oral issues. Furthermore, the utilization of oral services, such as treatment, remains remarkably low, despite the availability of long-term and favorable health insurance policies. The utilization of oral health services among college students is influenced by various factors, including residing in rural areas, consuming sugary beverages on a daily basis, brushing teeth at least twice a day, and practicing flossing.

## Introduction

Oral health is inseparable from human health and is considered as one of the ten standards of human health by the World Health Organization (WHO). WHO also establishes the standards of oral health: clean teeth, no cavities, no pain, normal gum color, no bleeding [1]. Poor oral health not only leads to oral diseases such as periodontitis, tooth decay, and tooth infection and loss, evidence in recent decades has also showed increased risks of systematic health problems such as diabetes, dementia, rectal cancer, atherosclerosis, pneumonia, and encephalitis from poor oral health [2–7].

In China, great attention seems to be attached to oral health. The government designates September 20 every year as “National Love Teeth Day“ [8]. And since 1983, a national oral health epidemiological survey has been conducted about every 10 years with a total of 4 surveys since then. The results of the Fourth National Oral Health Survey (2015–2016) showed that although the overall oral health status and behaviors across the country in different ages has gradually improved, the caries in children and periodontal condition in middle-aged adults have increased to different degrees [9].

However, there are still some problems with people’s dietary structure and eating habits, and they are changing worse rapidly with China’s continuous high economic growth for up to three decades. One of the most obvious issues is the high-sugar diet. Due to the development of the economy and society, the cost of sugar production has decreased and the consumption of sugar has increased linearly [10]. The big food corporations play a crucial role, by manipulating the market food supply, funding scientific studies in their favor, creating the illusion of low risk of harmful food, and inducing the population to take an unhealthy diet with high intake of sugar and high-fat foods. This not only leads directly to the coexistence of obesity and malnutrition in the population [11], but also to oral health-related problems. The total incidence rate of diabetes and pre diabetes in China has exceeded 50% [12], indicating a probable high threat to oral health which is also related to high-sugar diet.

Young people now face the temptation of a wide variety of snacks and beverages, and unhealthy oral health habits, which poses a significant risk of oral conditions to this group. A survey of college students from 26 low-, middle- and high-income countries in Asia, Africa and the Americas showed that 32.8% did not brush enough or did not brush their teeth, and 58.2% rarely or never went for dental checkups [13]. In a county in Norway, 47.8% of the young people, 68.1% of the middle-aged people and 74.2% of elder people visited ≥ 2 times every year from 2013 to 2014 [14]. While a survey conducted in a community in Beijing in 2011 found that the dental service utilization rate within one year among the young, middle-aged and elder people was only 16.5%, 10.3% and 9.6%, respectively [15]. Optimizing and promoting the use of oral health resources and improving national oral health is not an easy task, which has been a public health problem in China and across the world. In recent years, more and more attention has been paid to oral health and service utilization in China, and a large number of studies in China have focused on the oral health status and services of children, middle-aged people and the elderly [15–17]. For China, however, only the second national oral health epidemiological survey carried out in 1995 involved young people (aged 18 years), which showed a DMFT of 50.52% for male and 60.11% for female with only 34.27% of oral care utilization rate [18]. According to the NHANES III survey conducted in the United States, the DMFT among adolescents aged 16–19 years was 66.53% for males and 68.93% for females.The utilization rate of oral health services within one year was reported to be 73.95%. In comparison to the United States, China faces a significant disparity between the demand for oral health services and their actual utilization [19]. Since then few large surveys have provided evidence for oral health of this population. The oral health status and services of the youth and college students have been ignored for decades, remaining the associated factors among this population uncertain.

A series of theoretical models have been proposed to help understand health seeking related behaviors, and to propose strategies for behavioral change, like Theory of Planned Behavior (TPB), Health Belief Model (HBM), and Knowledge, Attitude/Belief, Practice (KAP) and Andersen Behavioral Model. The Andersen Behavioral Model is widely used and it describes the interrelationship between population characteristics (predisposing factors, enabling factors and need factors, PEN), health behaviors and health outcomes [20–22]. Herkrath’s Investigation of Oral Service Utilization among Adults in Rural and Urban Areas of Brazil Using the Anderson Model examines the factors influencing oral health service utilization. The study considers age, sex, education, and social networks as predisposing factors, while durable goods, household crowding, health insurance, and registration in primary health care [PHC] are viewed as enabling factors. Eating difficulties and tooth loss, as well as self-perception, are considered as needs factors. The research findings indicate that gender and age significantly influence the utilization of oral health services [23]. Nelson conducted a study to examine the factors influencing the utilization of children’s oral health services, based on the Anderson model. The study identified several influential factors, including children’s oral anxiety and fear (predisposing factors), having oral insurance (enabling factors), and parental values and attitudes. These findings highlight the importance of considering these factors when designing interventions to improve children’s oral health outcomes [24]. Xu [25] conducted a study comparing two populations in China: individuals aged 35–44 and 65–74 years old. The study construct an Andersonian model that considered sex, education, and belief as predisposing factors, household income and medical insurance as enabling factors, and perceived oral health, carious status, and number of teeth as needs factors. The findings revealed that perceived oral health status significantly influenced middle-aged individuals, while medical insurance and household income were important factors for older individuals. It is worth noting that health insurance has been consistently included as a significant predictor in both domestic and international studies utilizing the Anderson model, with a focus on children, middle-aged, and elderly populations.This study aimed to examine the factors that affect the utilization of oral health services among college students in southern China. The Andersen behavioral model was employed as a framework for this investigation. Specifically, our study focused on the impact of predisposing factors and needs factors on the utilization of oral health services. We did not analyze certain enabling factors such as the availability of medical resources and the price of medical services, as college students in this area have the same health insurance policy in China.Chinese college students are all covered by the Resident Basic Medical Insurance, and they usually go to local public dental specialized hospitals or dental departments of comprehensive hospitals, both of which are mainly public institutions and the prices are regulated by the local governments. In our study, the college students have good access to dental services because their university has an affiliated dental hospital located not far, and five other municipal level comprehensive hospitals providing dental care. The results would identify the patterns and influencing factors of oral health service utilization among college students, and provide evidence for oral health education and behavioral interventions in Chinese young people.

## Method

A cross-sectional survey was conducted on college students at a selected comprehensive university, Ganzhou City, Jiangxi Province of southern China. According to the grade structure of the medical school freshmen(Age:17–20), third(Age:20–23) and fifth grade(Age:23–25) were taken. The sample size was estimated according to the formula: *n* = *pq*/(*d*/*Z*_(1−*α*)/2_)^2^*=Z*^2^(_1−*α*)/2_*pq/*d*^2^. *p*: Estimated proportion of the population *q*: 1-*p*, estimated proportion of individuals in the population without a certain characteristic. d: Margin of error. Z: Z value for the normal distribution α: Significance level. Let *d* be a fraction of *p*, and when *d* = 0.15*p*, and when *α* = 0.05, *Z*_(1−*α*)/2_=1.96 ≈ 2, then *n* = 178*q/*p*. Based on the oral care utilization rate of 34.27% from the 4th National Oral Health Survey in the Mainland of China (2015–2016) [26], the required sample size should be as big as 342. In this study, considering the probability of refusal and missing data, we finally increased the required sample size to at least 600.

We stratify the survey participants into three groups based on their academic year: freshman, junior, and fifth year. We randomly sample individuals within each group and conduct the survey during evening study sessions or during breaks between classes. A total of 500 paper questionnaires were distributed, out of which 481 were responded to. Additionally, 206 questionnaires were distributed online, with 197 responses received. The overall response rate for the survey was calculated to be 96.03%. Prior to the survey, we present them with an informed consent form and obtain their signature. Totally 678 students participated in the survey, of which 481 received paper questionnaires and 197 responded electronic questionnaires. Those with incomplete answer(s), or those electronic questionnaire with a response time less than 1 min were regarded as invalid questionnaires. Finally 16 electronic questionnaires and no paper questionnaires were excluded, remaining 662 valid questionnaires in total. Considering the research power, we calculated the research power based on the sample size (*n* = 662), predictors (20), and the research power through the R software, which resulted in a research power of 1 for this study.

The questionnaire was designed according to the questionnaire of the Fourth National Oral Health Epidemiological Survey of China [9] as well as the Manual of Oral Health Behavior of Chinese Residents for Medical and Nursing Staff [27]. The final questionnaire was determined after a pilot study to test the reliability and validity.

The questionnaire survey was conducted in three aspects: predisposing factors, enabling factors and need factors, with the aim of exploring the factors associated with the utilization of oral health services by college students, with the dependent variable being whether or not they had used oral health services in the past 12 months [25].

The main predisposing factors were gender, mother’s education level, Father’s education level and knowledge, in demographic data belief and behaviors regarding oral health status. Mother’s education level and father education level was classified as low (junior high school and below), medium (high school, junior college, technical school, etc.), and high (college and above). However, in a large body of research, it has been found that mothers have a significant impact on their children’s oral health [28, 29]. Taking into account the issue of collinearity, our model includes the variable of mother education level. Knowledge, belief and behavior were measured by the variables of beliefs (oral health attitude and knowledge score), dessert consumption (desserts and sweets, sugary drinks, sweetened coffee, milk, etc.), and oral hygiene habits (brushing frequency, brushing duration, flossing frequency, and usage of fluoride toothpaste).

Health beliefs refer to an individual’s perception of health and disease, including their understanding of the severity and susceptibility to diseases, the impact of preventive measures, and the obstacles to adopting these measures [30].

The measurement of beliefs was conducted through four questions on attitudes towards oral health and eight questions on knowledge of oral health, which were derived from the Fourth National Oral Health Epidemiological Survey questionnaire [9]. For knowledge-related questions, a correct answer received one point, while an incorrect answer did not. For attitude-related questions, a positive attitude was scored as “agree”, while other responses such as “disagree”, “neutral”, and “don’t know” received zero. The total score for knowledge and attitudes was the sum of the 12 questions. For better statistical analysis, we divided the participants into two groups: “knowledge and belief > 8 points” and “knowledge and belief ≤ 8 points”.The enabling factors include residence, living cost and only-child. The residence was divided into urban and rural based on current family residence. Living costs was divided into low (<1000/month), medium (1000–1500/month) and high (>1500/month) levels. Only-child status was divided into yes and no.

The need factors were self-evaluation of oral health status, caries, ulceration, oral bleeding, dental defects, halitosis, calculus, and concern for oral health. Self- evaluation of oral health status included three levels: good, fair and poor. Caries, ulceration, oral bleeding, dental defects, halitosis, and calculus were self-reported and all classified into two levels: yes or no. The concern for oral health was divided into three levels: very concerned, fair, or not concerned.

Chi-square tests were performed to compare the utilization rates in the past 12 months among the respondents divided by different categorical variables.

Then logistic regression model analysis was conducted based on the predisposing factors, enabling factors, and need factors. In the first step, logistic model analysis was conducted for the predisposing factors to analyze the relationship between each variable on the outcome variable; in the second step, the enabling factor was added to the logistic model for analysis; in the third step, predisposing factors, enabling factors and need factors that met the criteria were included into the logistic model for analysis. The odds ratio (OR) and 95%CI were reported. Two-tailed tests were performed at the 0.05 level unless otherwise specified.

The responses of the questionnaires were double-entered using Epidata 3.1 software with consistency checks. All data were processed based on R version 4.2.1.

## Results

Table 1 showed the described demographic characteristics of college students. Among the 662 respondents, 49.40% of males; 50.60% of females, 80.06% of medical subjects and 19.94% of non-medical subjects(Science and Engineering: 26, Law: 41, Management: 28, Biology: 37); 31.0% of freshmen, 40.2% of third grade students, and 28.9% of fifth grade students; 44.8% of persons lived in urban areas and 55.2% lived in rural areas, and the education level of fathers is mainly middle and higher education level, accounting for 78.6%; the education level of mothers is mainly middle and lower education level, accounting for 75.5%.

**Table 1:** Demographic characteristics of the college students

Variables		***n***	%
Gender			
	Male	327	49.4
	Female	335	50.6
Grade			
	Freshman	205	31.0
	Third grade	266	40.2
	Fifth grade	191	28.9
Family address			
	Urban	296	44.8
	Rural	364	55.2
	Missing	2	0.2
Medical student			
	Yes	530	80.06
	No	132	19.94
Father’s education level			
	Low	140	21.4
	Medium	259	39.6
	High	255	39.0
	Missing	8	1.2
Mother’s education level			
	Low	262	40.2
	Medium	230	35.3
	High	160	24.5
	Missing	10	1.5

In the sample, 49.5% had used oral health services in the past, but only 30.2% had used oral health services in the past 12 months. The main reason for oral health service utilization in the past was treatment (59.6%) and only 12.8% were preventive.

Table 2 showed the oral health self-evaluation. In the evolution of oral health status, the oral health status was mainly fair (51.6%); 39% of people have oral issues, among which dental caries (25.7%) and oral bleeding (22.2%) are the main problems.

**Table 2:** Oral health status in college students

Variables		***n***	%
General oral health status			
	Good	224	33.9
	Fair	341	51.6
	Poor	96	14.5
	Missing	1	
Toothache in the past 12 months			
	Frequently	30	5
	Occasionally	338	55.8
	Never	238	39.3
	Missing(unknow)	56	
At least one oral problems			
	Yes	258	39.0
	No	404	61.0
Caries			
	Yes	170	25.7
	No	492	74.3
Ulceration			
	Yes	69	10.4
	No	593	89.6
Oral bleeding			
	Yes	147	22.2
	No	515	77.8
Dental defects			
	Yes	28	4.2
	No	634	95.8
Halitosis			
	Yes	54	8.2
	No	608	91.8
Calculus			
	Yes	109	16.5
	No	553	83.5

The description of college students’ knowledge, beliefs and behaviors about oral health is presented through Table 3. 84.1% had beliefs (knowledge + attitudes) > 8 scores; 76.4% brushed their teeth > = 2 / day; 92.6% brush duration > 1 min; 93.5% flossed infrequently; Most consumed desserts and sweets (62.8%), sugary drinks (50.6%) and sweetened coffee and milk (62.7%) .

**Table 3:** Oral health knowledge, beliefs and behaviors in college students

Variables		***n***	%
**Beliefs**			
Knowledge + Attitude and Belief			
	<=8	105	15.9
	> 8	556	84.1
**Behavior**			
Brushing frequency			
	< 2 /day	156	23.6
	>=2 /day	505	76.4
	Missing	1	
Brushing duration			
	< 1 min	48	7.4
	>=1 min	602	92.6
	Missing	12	
Flossing frequency			
	Infrequent use	618	93.5
	Frequently use	43	6.5
	Missing	1	
Dessert and candy consumption			
	Infrequent consumption	246	37.2
	frequent consumption	416	62.8
Sugary drinks consumption			
	Infrequent consumption	327	49.4
	frequent consumption	335	50.6
Sweetened coffee or milk consumption			
	Infrequent consumption	247	37.3
	frequent consumption	415	62.7

Table 4 presented the utilization rates of oral health care in the past 12 months. The predisposing factors gender, beliefs, frequency of brushing, frequency of flossing, consumption of desserts and sweets, consumption of sugary drinks, were statistically significant. All of the enabling factors were statistically significant; among the need factors, concern for oral cavity, and presence of dental caries were statistically significant.

**Table 4:** Utilization rate of oral health care in the past 12 months

Variables	***X*** ^**2**^	***df***	***P***
**Predisposing factors**			
Gender	4	2	0.045
Grade	4	2	0.134
Mother’s education level	3	3	0.264
Father’s education level	1.2	2	0.537
Studying subject	0.15	1	0.691
Toothache in the past 12 months	31	2	< 0.001
Beliefs	9	2	0.003
Brushing frequency	16	2	< 0.001
Brushing time	0.3	2	0.597
Flossing frequency	10	2	0.002
Use of fluoride toothpaste	0.2	1	0.634
Dessert and candy consumption	10	2	0.001
Sugary drinks consumption	29	2	< 0.001
Sweetened coffee or milk consumption	0.4	2	0.526
**Enabling Factors**			
Rural or urban	19	2	< 0.001
Only child	5	2	0.022
Living costs	13	3	0.002
**Need factors**			
Concern for the oral health	32	3	< 0.001
Caries	29	2	< 0.001
Ulceration	1	2	0.286
Oral bleeding	0.08	2	0.773
Dental defects	4	2	0.056
Halitosis	0.2	2	0.684
Calculus	3	2	0.106
Oral health status	1	3	0.552

Table 5 are the results from the hierarchical logistic regression models for the predisposing, enabling, and need factors of using oral health service in a year. Both model 1 with predisposing factors and model 2 with added facilitators were associated with utilization of oral health services. In the final model 3, better beliefs (*OR* = 1.84, 95% *CI*:=1.02–3.43) and frequent consumption of sugary drinks (*OR* = 2.90, 95% *CI*:=1.90–4.47), brushing frequency > = 2 times per day (*OR* = 2.09, 95% *CI* = 1.24–3.61), flossed frequently (*OR* = 2.63, 95% *CI* = 1.21–5.76), had dental caries (*OR* = 2.07, 95% *CI* = 1.35–3.17) used oral health services more; lived in rural areas (*OR*:0.52, 95% *CI* = 0.34–0.80) and had an oral concern (*OR* = 0.48, 95% *CI* = 0.31–0.74) or no concern (*OR* = 0.26, 95% *CI* = 0.08–0.67) utilized oral health services less.

**Table 5:** Logistic regression of oral health service utilization performed in the past 12 months

Variables	Model 1	Model 2	Model 3
	OR (95%CI)	OR (95%CI)	OR (95%CI)
**Predisposing factors**			
Gender (male)	0.72(0.49, 1.05)	0.72(0.484, 1.06)	0.75(0.49, 1.12)
Mother’s education (Low)			
Medium	1.13 (0.74, 1.73)	0.93(0.60, 1.45)	0.89(0.56, 1.40)
High	1.29 (0.81, 2.04)	0.83(0.48, 1.42)	0.78(0.44, 1.36)
Beliefs			
Knowledge, attitude and belief score > 8 (≤ 8)	2.21(1.27, 4.03)^**^	2.04(1.16, 3.73)^*^	1.84(1.02, 3.43)^*^
Frequent dessert and candy consumption (< 2/day)	1.58(1.04, 2.40) ^*^	1.52(0.99, 2.34)	1.44(0.94, 2.25)
Frequent sugary drinks consumption (< 2/day)	3.02(2.02, 4.55)^***^	2.93(1.95, 4.45)^***^	2.90(1.90, 4.47)^***^
Frequent sweetened coffee or milk consumption (< 2/day)	0.72(0.48, 1.07)	0.73(0.48, 1.10)	0.73(0.47, 1.11)
Brushing frequency ≥ 2 /day (< 2/day)	2.38(1.46, 4.01)^***^	2.41(1.46, 4.09)^***^	2.09(1.24, 3.61)^**^
Brushing duration ≥ 1 min (< 1 min)	1.25(0.63, 2.63)	1.31(0.64, 2.81)	1.15(0.55, 2.53)
Frequent flossing using (No)	3.02(1.50, 6.14)^**^	3.22(1.54, 6.82)^**^	2.63(1.21, 5.76)^*^
Use of fluoride toothpaste (No)	0.88(0.60, 1.30)	0.89(0.60, 1.32)	0.81(0.54, 1.22)
**Enabling Factors**			
Rural (Urban)		0.53(0.35, 0.81)^**^	0.52(0.34, 0.80)^**^
Living costs (No)			
Medium		1.67(0.96, 2.96)	1.60(0.92, 2.86)
High		1.66(0.84, 3.33)	1.53(0.76, 3.15)
Only-child (No)		1.20(0.72, 1.99)	1.14(0.67, 1.93)
**Need factors**			
Oral health status (good)			
Fair			0.92(0.60, 1.41)
Poor			0.64(0.34, 1.21)
Caries (No)			2.07(1.35, 3.17)^***^
Concern for the oral health (very concern)			
Fair			0.48(0.31, 0.74)^***^
No			0.26(0.08, 0.67)^**^

Model 1: The predisposing factors were included in the logistic model for analysis

Model 2: The predisposing factors and enabling factors were included in the logistic model for analysis

Model 3: All variables were included in the logistic model analysis

*P < 0.05, **P < 0.01, ***P < 0.001

## Discussion

This study explored the factors associated with oral health service utilization among college students on the basis of Andersen’s behavioral model. The utilization of dental services by this group of college students has rarely been involved in current research, and this is an important point to add to this study.

The results showed that 30.2% of the college students went for oral health services during the past 12 months at least once. In Si-Si Peng’s study [31], the utilization rate of oral health services among college students in the past 12 months was 34%, which is similar to the results of this study. According to the Fourth National Oral Health Epidemiological Survey Report, the utilization rate of oral health services for adults aged 35–44 years in the past 12 months is 21.4%, and the utilization rate of oral health services for the elderly aged 65–74 years is 20.7% [9] The past 12-month oral health service utilization rates in a Brazilian study [32] were 59.3%, 55.5%, 54.7, 51%, and 36.5% for ages 18–21, 22–34, 35–44, 45–64, and 65 + years, respectively. It seems that the utilization rate of oral health services reduces with the increase of age [33].

In addition, the utilization of oral health services by college students was treatment-oriented rather than prevention-oriented, which is consistent with previous studies [25, 34–36]. In the present study, three-fifths of the students visited the dentist for treatment, higher than children (47.2%) [37] and lower than middle-aged (78.7%) and elderly (93.7%)) [25]. This is much higher than the reporting rate for young people in developed countries (26.6%) [38]. In recent years, the inadequacy of white teeth and untidy teeth have become the main problems of dental consumers in China, in addition to dental caries, and the younger consumption and oral “beauty” have become the “new” characteristics of oral health service consumption. From the results of this study, the low consultation rate of young people’s oral health and the high demand are still extremely mismatched, which may greatly restrict the oral service industry development.

About two in five (39%) of college students reported having at least one oral health problem, which was closely approximate to the self-reported rates of oral health problems among middle-aged adults (36.1%) [39] and older adults (42.68%) [40]. The most commonly reported periodontal disease was dental caries (25.7%), and the number was similar to that from another study carried out in northern China [41]. The reporting rates of periodontal diseases such as oral bleeding and dental calculus were 22.2% and 16.5%, respectively. The rates are much lower than the findings of the 4th National Oral Health Epidemiological Survey for children aged 15 (64.7%, 73.6%) and people aged 35–44 (87.4%, 96.7%), which were clinically examined according to the procedures and criteria recommended by WHO [1]. Some clinical data from hospitals indicated that the prevalence of periodontal diseases were also high among young people in China, with a 72.2% detection rate of dental calculus and 51.6% detection rate of dental bleeding among people aged 18–24 years [42]. The results from the same study showed that the detection rates of caries (30.1%) and defects (5.1%) from clinical tests were close to those from our self-assessed rates. Those findings indicated an underestimation of dental disease prevalence from self-reported studies in China, especially when assessing periodontal diseases. Compared with New Zealand adults aged ≥ 35, Chinese reported many clinical oral diseases similarly, while they were more likely to underestimate bleeding [43]. The awareness of dental health and periodontal diseases among young Chinese remained very poor which is highly detrimental to prevention and service utilization of oral health.

Among the predisposing factors, most people had good oral beliefs (84.1%) and good oral health care habits, but did not pay as much attention to oral health protection in their daily diet, with 62.8%, 50.6%, and 62.7% consuming desserts and sweets, sugary drinks, and sweetened coffee and milk on a daily basis. In the bivariate analysis of sweets, the consumption of desserts and/or sweets, and the consumption of sugary drinks were all significant, while only sugary drinks were significant in the logistic regression model. Foods with sweet taste in nature provide energy for physical activities, and out of instinct, humans have a strong taste for sweetness. However, with the continuous intake of sweets comes some health risks, Pujara research revealed a significant association between the consumption of sweets and the risk of tooth decay and periodontal diseases [44]. A systematic review on the consumption of sugary drinks and oral health indicated that the consumption of sugary drinks significantly increases the risk of tooth decay and tooth erosion. Furthermore, there is a dose-response relationship between the consumption of sugary drinks and the incidence of tooth decay and tooth erosion [45]. Thus it would like to indirectly promote oral health service utilization among college students. The Dietary Nutrient Reference Intake for Chinese Residents [46] recommends that adults should not exceed 50 g/day of added sugar, and the Healthy China Initiative (2019–2030) [47] advocates that the daily per capita intake of added sugar should be reduced to less than 25 g by 2030.

It has long been the general trend that the Chinese are not sugar-obsessed relative to the majority of the region, and FAO data revealed that China’s per capita sugar consumption was at a relatively low level in the global ranking of countries in 2017, roughly 1/3 or less than that of developed countries such as the United States (126.4 g) Australia (95.6 g), Canada (94.9 g), and New Zealand (90.9 g) [48]. However, this favorable diet is being severely challenged in recent years. According to the survey results from CHNS 1997–2011, the total sugar intake for Chinese children and adolescents aged 3–17 increased from 11.2 ± 0.3 g/day to 28.1 ± 0.5 g/day, with added sugar increasing from 1.0 ± 0.1 g/day to 7.2 ± 0.3 g/day [49]. The sugar intake among Chinese children and adolescents has been steadily increasing. In our study, 62.8% of college students consume desserts and sweets every day, and 50.6% of college students consume sugary drinks daily, which is significantly higher than the results from a study in Saudi Arabia [50] (34.4% of participants consume chocolates and candies daily, and 34.8% consume carbonated drinks daily).Our sugar consumption continues to grow, ranking third in the world after India and the EU [51]. Commercial interests group induce or kidnap population to build unhealthy diet cultures and behaviors in order to expand their own profits. For instance, milk tea and fruit tea is labeled as “young”, “fashionable” and even “life-saving beverage” these years, thereby strengthening the reason for consumption among Chinese young people. China’s beverage unicorn Nayuki Tea & Bakery showed that about 30% of consumers in China spend over 400 yuan ($61) on the beverage every month, and millennials with a higher education background were the primary customers. The retail market size of Chinese desserts reached 93.7 billion yuan and the market size of sugary drinks reached 369 billion yuan in 2019 [52, 53]. While traditional health drinks such as tea and pure coffee are greatly impacted. In urban population, 42.1% of free sugar intake comes from sugary drinks and dairy beverages, and the percentage is even higher among children and adolescents [54]. The large size of the sweets market means that there is a huge population in the midst of exposure, which has a great impact on people’s oral health. Milk tea stores are everywhere in this country, and people are more likely to be exposed to sugary drinks leading to oral health risks. It is urgent to take action for beverage and food companies to reduce sugar in their products, and every consumer should also reinforce the concept of sugar-reduced diet.

We found that good oral beliefs promote the use of oral health services [55]. Also, personal oral hygiene behaviors have a strong relationship with oral health service utilization; studies have shown that good oral hygiene beliefs create good oral hygiene habits [56, 57]. The study showed that people who have good oral hygiene habits such as brushing and flossing regularly have better oral health status [58]. The study shows that people with good oral hygiene practices such as brushing and flossing regularly have better oral health, are able to notice early oral problems and access dental treatment earlier in their oral care routine [25]. The oral hygiene beliefs create better oral health.

Among the contributing factors, living in a rural area hindered people’s utilization of oral health services. This is similar to the results of previous studies [59–61]. This may be related to the unequal distribution of income and oral resources between urban and rural areas [62–64]. Being an only child and cost of living were not significant in the logistic regression multivariate analysis with oral health service utilization. This may be because Chinese college students, upon entering college life, are not given excessive living expenses by their parents in order for them to get enough exercise, regardless of whether they are only children themselves, and the cost of living for college students is basically the same [65] which makes it not statistically different.

Among the need factors, people with dental caries would increase oral health service utilization. This is closely related to our problem-oriented treatment model [25, 34–36], where most people seek dental treatment only after finding out that they have acquired caries [66–68]. People with average and no concern for oral hygiene are less likely to utilize oral health services. People who have little concern for oral hygiene do not usually pay attention to their oral problems. This also leads to low sensitivity of individuals when early oral problems appear and reduces the reliance on dental clinics. This study used a self-assessed approach to evaluating oral health status, which is widely used in epidemiological surveys on oral health. Besides, Thomson’s study [43] carried in middle-aged and elder adults from China and New Zealand has showed that there is not very large difference between self-reported oral health status and clinical oral diseases, and the two assessment results are consistent in most aspects of clinical oral diseases. As Chinese population rarely have regular oral examinations and most people could not know their actual dental conditions, and people’s medical visits are mostly based on the awareness rather than the actual status of their own oral health, the self-evaluation oral health could be suitable for investigation potential risk factors of dental care utilization.

There are still some shortcomings in this study. Firstly, the data are cross-sectional data obtained from sampling at one university, and caution is needed regarding the extrapolation of the results. Secondly, the data were sampled by convenience sampling with some selection bias. Thirdly, the study only elaborated on oral health service utilization at the individual level and did not include public health policies for analysis, which is an area for subsequent improvement. Finally, the repondents’ dental health in our survey was self-reported, and future research if possible should use professional dental examinations to assess accurate oral health of the subjects and evaluate the reliability of self-assessment methods when needed.

## Conclusion

Chinese college students demonstrate some knowledge and attitudes towards oral health. However, they tend to neglect oral hygiene and have limited understanding of their own oral issues. Furthermore, the utilization of oral services, such as treatment, remains remarkably low, despite the availability of long-term and favorable health insurance policies. The utilization of oral health services among college students is influenced by various factors, including residing in rural areas, consuming sugary beverages on a daily basis, brushing teeth at least twice a day, and practicing flossing. Unfortunately, the prevalence of these two simple yet effective oral care behaviors is still inadequate among college students, particularly the latter. Considering the current poor oral health status observed in other age groups, such as children and older adults, it is strongly recommended to promote oral health education and provide guidance to all populations. By popularizing these simple and effective oral care behaviors, the Chinese population can enjoy lifelong benefits.

## Data Availability

The data that support the findings of this study are available from the corresponding author upon reasonable request.
